# Altered Cytokine Expression and Barrier Properties after In Vitro Infection of Porcine Epithelial Cells with Enterotoxigenic *Escherichia coli* and Probiotic *Enterococcus faecium*

**DOI:** 10.1155/2017/2748192

**Published:** 2017-05-14

**Authors:** Martina Kern, Dorothee Günzel, Jörg R. Aschenbach, Karsten Tedin, Angelika Bondzio, Ulrike Lodemann

**Affiliations:** ^1^Institute of Veterinary Physiology, Department of Veterinary Medicine, Freie Universität Berlin, Oertzenweg 19b, 14163 Berlin, Germany; ^2^Institute of Clinical Physiology, Charité—Universitätsmedizin Berlin, Campus Benjamin Franklin, Hindenburgdamm 30, 12203 Berlin, Germany; ^3^Institute of Microbiology and Epizootics, Department of Veterinary Medicine, Freie Universität Berlin, Robert-von-Ostertag-Str. 7-13, 14163 Berlin, Germany; ^4^Institute of Veterinary Biochemistry, Department of Veterinary Medicine, Freie Universität Berlin, Oertzenweg 19b, 14163 Berlin, Germany

## Abstract

The aim of the present study was to elucidate the effects of the probiotic feed additive *Enterococcus faecium* NCIMB 10415 (*E. faecium*) on porcine jejunal epithelial cells (IPEC-J2) during an in vitro challenge with enterotoxigenic *Escherichia coli* (ETEC). Cells were incubated with *E. faecium*, ETEC, or both, and the effects on barrier function and structure and intra- and intercellular signaling were determined. Coincubation with *E. faecium* abolished the ETEC-induced decrease in transepithelial resistance (*R*_t_) (*p* ≤ 0.05). No differences were seen in the expression levels of the intercellular connecting tight junction proteins examined. However, for the first time, a reorganization of the monolayer was observed in ETEC-infected cells but not in coincubated cells. ETEC induced an increase in cytotoxicity that was prevented by coincubation (*p* ≤ 0.05), whereas apoptosis rates were not affected by bacterial treatment. ETEC increased the mRNA expression and release of proinflammatory cytokines TNF-*α*, IL-1*α*, and IL-6 which could be prevented by coincubation for TNF-*α* mRNA expression and IL-6 protein (*p* ≤ 0.05). Likewise, cAMP concentrations elevated by ETEC were reduced in coincubated cells (*p* ≤ 0.05). These findings indicate a protective effect of the probiotic *E. faecium* on inflammatory responses during infection with ETEC.

## 1. Introduction

The gut microbiota has an important impact on immune responses and barrier function of the intestinal mucosa and, thereby, influences overall gut health. Several recent studies tried to assess the complex cross talk between microbiota, intestinal barrier, immune system, and the gut-brain axis [1, 2]. The high relevance of such cross talk has been shown for various disease phenotypes such as inflammatory bowel disease, which can be attributed, in part, to perturbations in the composition of the intestinal microbiota [3, 4]. To support the establishment or restoration of a healthy gut microbiota, probiotics have been proposed as preventive and therapeutic measures [3]. They have been successfully applied as a therapy in dysbiosis-associated diseases relevant in human medicine such as ulcerative colitis [5]. In animal rearing, probiotics have become valuable tools to prevent diarrhea in critical production phases and, thereby, exert positive effects on the performance and health status [6–8].

A very critical period in piglet rearing is weaning where diarrhea attributable to infections with enterotoxigenic *Escherichia coli* (ETEC) represents a major problem [9–11]. The probiotic strain *Enterococccus faecium* NCIMB 10415 (*E. faecium*), used in the present study, is a licensed feed additive for piglets and sows in the EU, and its effects on diarrhea incidence and performance have been studied in various feeding trials [7, 12, 13]. It has been found to reduce diarrhea incidence in piglets in the pre- and postweaning period [7, 13], and when applied to sows, it diminishes piglet loss after birth [13]. It has further been shown to affect gut immunology [12, 14]. In a previous study, in vitro coincubation with *E. faecium* reduced the decrease in transepithelial resistance (*R*_t_) induced by ETEC in human colonic and porcine jejunal epithelial cells [15]. The focus of the present study was to examine the effects of *E. faecium* on barrier properties and immunological readout values in a porcine intestinal epithelial cell model in vitro.

The structural barrier of the intestinal epithelium is formed by the inner lining of intestinal epithelial cells, which are connected by intercellular junctions. The paracellular permeability is regulated by tight junction (TJ) structures. Claudins are the main components of tight junctions. Some of these proteins, such as claudin-1, claudin-3, and claudin-5, have sealing functions, whereas others form channels with selectivity for cations or anions or are permeable to water [16]. TJ proteins and their localization have been shown to be regulated by cytokines [17], for example, by inducing the redistribution of various TJ proteins via internalization [18] or by regulating the transcription level of TJ proteins [19].

Based on these prior observations, we examined whether the protective effects of *E. faecium* during an ETEC challenge are attributable to an enhancement of barrier function by modulating TJ protein expression and localization, the cell structure, or the vitality of the cells. Furthermore, it was hypothesized that *E. faecium* reduces the release of inflammatory cytokines during an ETEC challenge. As the heat-labile toxin of ETEC enhances cAMP production, which can regulate the expression of proinflammatory cytokines [20], we also assumed that the intracellular concentrations of cAMP during infection of host cells may be modulated by the probiotic.

## 2. Material and Methods

### 2.1. Cell Culture

Intestinal porcine epithelial cells (IPEC-J2) were kindly provided by Prof. Dr. Anthony Blikslager (North Carolina State University, USA). They were cultivated as described in Klingspor et al. [15] in DMEM-Ham's F12 medium (Biochrom, Berlin, Germany) supplemented with 5% fetal bovine serum (FBS, Biochrom), 2.5 mmol/l L-glutamine (Biochrom), 5 ng/ml epidermal growth factor (Biochrom) and ITS (insulin [5 *μ*g/ml], transferrin [5 *μ*g/ml], and selenium [5 ng/ml]; Sigma-Aldrich Chemie GmbH, Taufkirchen, Germany), and penicillin-streptomycin (100 units penicillin and 100 *μ*g streptomycin/ml, Sigma-Aldrich Chemie GmbH). Cells were split at a ratio of 1 : 3 every 7 days.

For experiments, cells were either seeded in 24-well cell culture plates (15 mm diameter, TPP, Faust Lab Science, Klettgau, Germany) or on cell culture inserts (Transwell Clear, 12 mm diameter, 0.4 *μ*m pore size [Corning B.V., Schiphol-Rijk, The Netherlands], Millicell cell-HA (12 mm diameter, 0.45 *μ*m pore size [Merck Millipore Ltd., Darmstadt, Germany]) collagenized with rat tail collagen I (Serva Electrophoresis GmbH, Heidelberg, Germany) at a concentration of 10^5^ cells per well or per cell culture insert. For apoptosis and cytotoxicity assays, cells were cultivated on 96-well cell culture plates (34 mm^2^, Lumox multiwell, Sarstedt, Nümbrecht, Germany) at a concentration of 10^4^ cells per well. Before being used in an experiment, the cells were cultivated for 14–21 days. On the day prior to experiments, the cells were supplied with serum- and antibiotic-free media.

### 2.2. Bacterial Strains

Enterotoxigenic *E. coli* IMT4818 (ETEC, isolated from a 2-week-old piglet with enteritis, O149:K91:K88 (F4), positive for the existence of virulence genes *est*-1a, *est*-2 [genes coding for heat stable enterotoxins I and II], and *elt*-1a/b [gene coding for heat labile enterotoxin I] by polymerase chain reaction [PCR]) was grown in LB medium (Luria/Miller) containing 10 g/l tryptone, 5 g/l yeast extract, and 10 g/l NaCl, at a pH of 7.0 (Roth, Karlsruhe, Germany).

The probiotic *E. faecium* NCIMB 10415 strain (from Cylactin^®^, DSM, Kaiseraugst, Switzerland) was cultivated in brain-heart infusion (BHI) broth (OXOID GmbH, Wesel, Germany). The bacteria were incubated overnight at 37°C and subcultured for approximately 180 min until midlog phase on a shaker at 37°C, centrifuged, and washed twice with phosphate-buffered saline (PBS, Biochrom). Bacterial cells were then resuspended in DMEM-Ham's F12 medium without penicillin/streptomycin or FBS. The concentration was adjusted to 10^8^ colony-forming units (CFU)/ml based on optical density (*E. faecium* OD = 0.9, 600 nm, ETEC OD = 1.3, 600 nm) measured in the Heilos™ Epsilon spectrophotometer (Thermo Scientific, Waltham, USA). The bacterial concentration was confirmed by serial dilution followed by determination of viable counts on LB-plates.

### 2.3. Incubation of Cells with Bacterial Strains

Bacteria (*E. faecium* or ETEC) were added to the apical side of cell culture inserts, 24-well plates, or 96-well plates. The intestinal cells were infected with 10^6^ bacteria per cell culture insert or well, corresponding to a multiplicity of infection (MOI) of approximately 10 bacteria per seeded cell. The 96-well cell culture plates were infected with 10^5^ bacteria per well, which also equates to an approximate MOI of 10. To test the effect of *E. faecium* on ETEC infection, the cells were coincubated with both ETEC and *E. faecium* strains. In the coincubation studies with the probiotic and the pathogenic strains, the cells were first preincubated with *E. faecium*, and the ETEC strain was added 2 h later. In the following, this setup will be called “coincubation.” The incubation times given in the legends were calculated based on the duration of the incubation with the ETEC strain. After 2 h of incubation with the respective strains, gentamycin (50 *μ*g/ml medium, Biochrom) was added to kill the bacteria as described in Klingspor et al. [15].

For analysis of cyclic adenosine monophosphate (cAMP), the incubation time of the respective strains was 4 h and samples were taken after 4 h of incubation.

**Figure 1:**
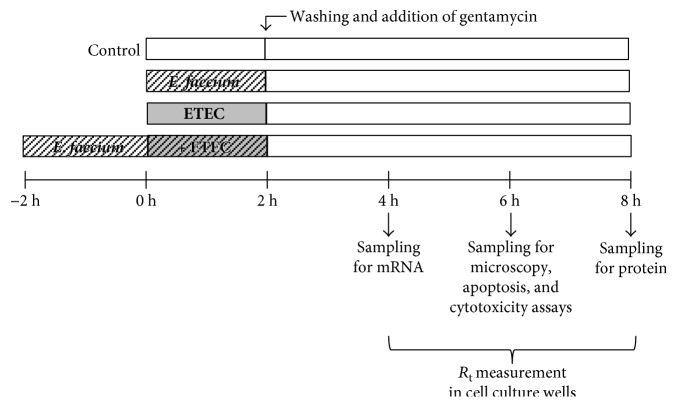
Experimental set-up.

For mRNA expression, protein expression, and confocal laser scanning microscopy (CLSM), samples were taken at various time points as indicated in Figure 1.

### 2.4. Resistance Measurements (*R*_t_)

*R*_t_ was determined for cells grown in cell culture inserts with a Millicell-ERS (Electrical Resistance System; Millipore GmbH, Schwalbach, Germany). The blank value (cell culture insert and medium without cells) was subtracted from the measurements, and the values were corrected for the membrane area. The *R*_t_ values before the beginning of experiments were 3509 ± 117 Ω · cm^2^ [mean ± SEM].

### 2.5. Real-Time Quantitative Polymerase Chain Reaction

Samples for analysis of mRNA expression were taken from 24-well plates. Cell layers were rinsed twice with PBS, and the cells were harvested in PBS with a cell scraper and centrifuged (5 min, 200 *g*). RNAlater (Qiagen GmbH, Hilden, Germany) was added to avoid RNAse degradation, and the samples were frozen at −20°C. Three wells of a culture plate were pooled per sample.

The following protocol is described in more detail by Lodemann et al. [21]. The nucleospin RNA II Kit (Macherey-Nagel GmbH & Co. KG, Düren, Germany) was used to isolate total RNA. The RNA concentration was quantified by NanoDrop (PEQLAB Biotechnologie GmbH, Erlangen, Germany). The RNA quality was determined with a 2100 Bioanalyzer (Agilent Technologies, Böblingen, Germany), and only samples with an RNA integrity number above 7 were included in further analyses. cDNA was synthesized with the iScript™ cDNA Synthesis Kit (Bio-Rad Laboratories GmbH, Munich, Germany) by reverse-transcribing 100 ng total RNA from the IPEC-J2 cells in a final volume of 200 *μ*l in an iCycler iQ™ Real-Time PCR Detection System (Bio-Rad Laboratories GmbH).

Information about primers for the targets is given in Table 1. Three reference genes were used for normalization (GAPDH, TBP, and YWHAZ) as described in Klingspor et al. [15]. Primers were synthesized by Eurofins MWG Synthesis GmbH (Ebersberg, Germany).

RT-qPCR was conducted in the iCycler iQ Real-Time PCR Detection System (Bio-Rad Laboratories GmbH) by using SYBR green I detection. The samples were analysed in duplicate; the final volume of the wells (25 *μ*l) contained iQ SYBR Green Supermix (Bio-Rad Laboratories GmbH), primers (0.5 *μ*l of 20 pmol/*μ*l each), and 5 *μ*l cDNA. Controls included transcription reactions without reverse transcriptase to prove the absence of genomic DNA contamination. iQ5 software was used for the analysis of the relative amount of the target genes (Bio-Rad Laboratories GmbH).

### 2.6. Enzyme-Linked Immunosorbent Assay (ELISA)

#### 2.6.1. IL-6

Supernatants of IPEC-J2 cells were collected from 24-well plates after 8 h (see Figure 1), centrifuged (5 min, 4000 *g*, 4°C), and frozen at −80°C until use. Porcine IL-6 ELISA (Raybiotech, Norcross, USA) was performed according to the manufacturers' instructions. Samples were analysed in duplicate.

#### 2.6.2. IL-1*α*

Cell-culture supernatants were harvested from 24-well plates after 8 h, centrifuged (5 min, 4000 *g*, 4°C), and frozen at −80°C until use. IL-1*α* concentrations were determined with the Pig Interleukin 1 *α* (IL-1*α*) ELISA Kit (Cusabio, Wuhan, China) according to the manufacturers' instructions. Assays were performed in duplicate.

#### 2.6.3. TNF-*α*

Supernatants of IPEC-J2 cells were taken from 24-well plates after 8 h, and cell debris was removed by centrifugation at 4000 *g* and 4°C. Samples were frozen at −80°C until use. Quantikine ELISA specific for porcine TNF-*α* (R&D systems, Minneapolis, United States) was performed according to the manufacturers' instructions. Assays were performed in duplicate. To enhance the protein content, the sample volume was increased to 250 *μ*l per well. Optical density was determined within 30 min by using a microplate reader (EnSpire, Multimode Plate Reader, Perkin Elmer, Rodgau, Germany). Readings at 540 nm were subtracted from the readings at 450 nm and blank corrected. A four parameter logistic (4-PL) curve fit was generated for the calculation of the results.

#### 2.6.4. cAMP

cAMP concentrations were determined by using the Cyclic AMP XP Assay Kit #4339 (Cell Signaling Technology, Danvers, United States). On the day prior to experiments, confluent 24-well plates of IPEC-J2 cells were washed twice with warm PBS and supplied with serum- and antibiotic-free media. On the experimental day, cells were lysed with Roche cOmplete™ Lysis-M (Sigma-Aldrich, Munich, Germany) after 4 h of bacterial treatment and centrifuged (5 min, 600 *g*, 4°C), and cAMP ELISA was performed following the manufacturers' instructions. Assays were performed in duplicate.

### 2.7. Apoptosis and Cytotoxicity Assays

Apoptosis and cytotoxicity were assessed at 6 h after the addition of the bacterial strains by using the ApoTox-Glo™ Triplex Assay (Promega, Madison, USA) according to the manufacturer's instructions. This assay measures apoptosis by quantifying caspase-3/7 and cytotoxicity by quantifying dead-cell protease. Excitation and emission settings for cytotoxicity measurements were set to excitation 485 nm and emission 520 nm. Apoptosis was determined by luminescence measurements.

### 2.8. Western Blots

Western blot (WB) analyses were performed by using standard techniques as described in detail by Amasheh et al. [23]. Primary and secondary antibodies are given in Table 2. The Lumi-LightPLUS Western Blotting Kit (Roche, Grenzach Wyhlen, Germany) was used to detect relevant protein bands via the Fusion FX 7 image acquisition system (Vilber Lourmat, Eberhardzell, Germany). Densitometric signal analysis was performed by using AIDA software (Raytest, Berlin, Germany), and *β*-actin was used as loading control.

### 2.9. Confocal Laser Scanning Microscopy

IPEC-J2 cells grown on membrane supports were washed with PBS with Ca^2+^ and Mg^2+^ and then incubated for 15–20 min in a 2% paraformaldehyde solution at room temperature. The paraformaldehyde was subsequently deactivated in a 125 mmol/l glycine solution. After subsequent rinsing with PBS containing Ca^2+^ and Mg^2+^, preparations were stored in PBS at 4°C.

For staining, cells were permeabilized in 0.5% Triton-X100 in PBS (10 min) and incubated with primary antibodies (1 : 200 in PBS, 4°C, overnight). After the cells had been thoroughly washed, incubation with secondary antibodies (Jackson ImmunoResearch, Newmarket, UK, Cy2 Fab goat anti-mouse, Cy5 Fab goat anti-rabbit) was carried out at a concentration of 1 : 600 together with DAPI (2-(4-amidinophenyl)-1H-indole-6-carboxamidine, final concentration 1 *μ*g/ml, for 45 min at room temperature, Roche Grenzach Wyhlen, Germany). Preparations were rinsed thoroughly and embedded in ProTaqs Mount Fluor (Biocyc, Luckenwalde, Germany). Confocal images were obtained with a confocal laser scanning microscope (Zeiss LSM780, Jena, Germany) by using excitation wavelengths of 405 nm, 488 nm, and 633 nm.

### 2.10. Calculations and Statistical Analysis

The statistical analyses and the plotting of graphs were carried out by means of the SPSS program for Windows, version 23 (Jandel, Chicago, IL, USA) and Microsoft Excel 2010 (Microsoft Corporation, Redmond, US).

The statistical significance of differences was assessed by variance analysis. For *R*_t_ values, the expression of cytokines, measurement of cAMP concentrations, and apoptosis and cytotoxicity assays, a variance analysis with the fixed factor “treatment” (“control,” “*E. faecium*,” “ETEC,” and “*E. faecium* + ETEC”) was conducted. If ANOVA indicated a significant difference, a post hoc Scheffé or least significant difference (LSD) test was conducted per time point to isolate the means that differed. For the protein expression of TJ proteins, an unpaired Student's *t-*test was used. The differences between groups were considered statistically significant for *p* ≤ 0.05. Statistical significance is marked in the figures using letter coding; means are not different if they share the same letter and are different if they do not share at least one common letter. Results are given as means ± SEM. At least three independent experiments were conducted, as indicated in the figure legends.

## 3. Results

### 3.1. *R*_t_

**Figure 2:**
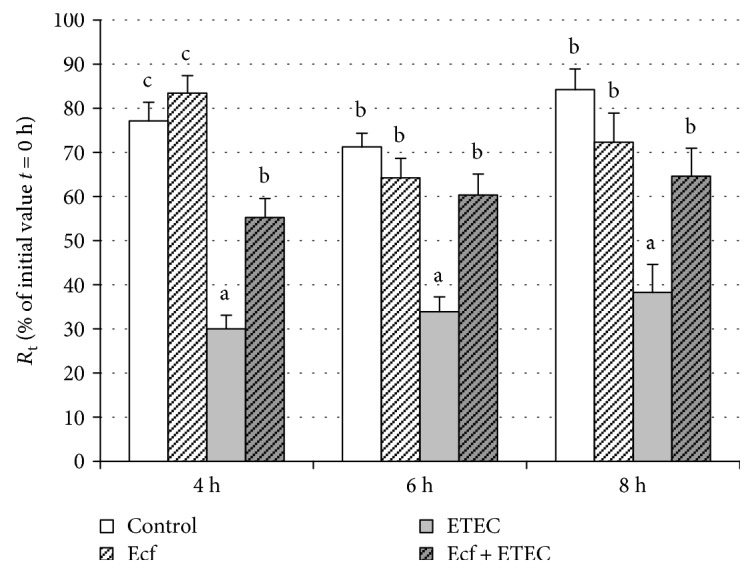
*R*_t_ in cells incubated with bacterial strains (Ecf, ETEC, and Ecf + ETEC) and in control cells not exposed to bacteria [means ± SEM]. Groups differ significantly (*P* ≤ 0.05) when marked with different letters, (e.g. a, b) in the figure. *N* = 5 independent experiments.

ETEC addition significantly reduced the *R*_t_ of IPEC-J2 cells (*p* ≤ 0.05). This decrease in *R*_t_ was reduced or even prevented by coincubation with *E. faecium* as indicated by measurements taken at 4 h, 6 h, and 8 h (*p* ≤ 0.05) (Figure 2).

### 3.2. Tight Junction Proteins

**Figure 3:**
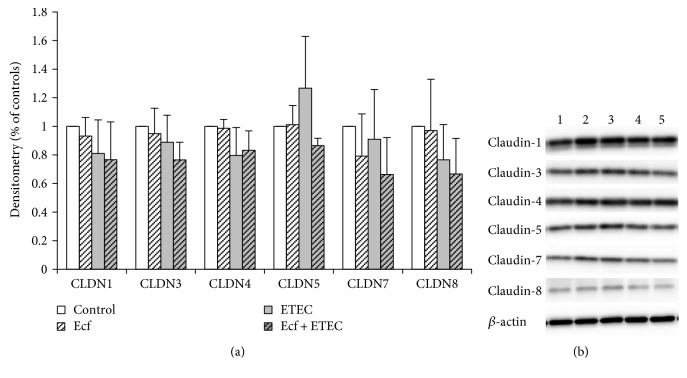
Protein expression (Western blot) of claudin-1, claudin-3, claudin-4, claudin-5, claudin-7, and claudin-8 after treatment with bacterial strains (Ecf, ETEC, and Ecf + ETEC). (a) Protein expression relative to respective controls [means ± SEM]. Samples were taken after 8 h. No differences were observed between treatment groups. *N* = 3 independent experiments per bar. (b) Exemplarily, data of one Western blot is shown. 1 = control (8 h), 2 = Ecf (8 h), 3 = ETEC (8 h), 4 = Ecf (8 h) + ETEC (6 h), and 5 = Ecf (10 h) + ETEC (8 h). Sample 4 was included as a control to rule out effects of longer total bacterial incubation (preincubation) and was not included in the statistical analysis shown in (a).

The changes in *R*_t_ indicated effects on barrier function. To elucidate whether these changes were attributable to the altered abundance of TJ proteins, we examined the expression of selected TJ proteins. However, in Western blots, no significant differences were detected in the expression of the TJ proteins claudin-1, claudin-3, claudin-4, claudin-5, claudin-7, or claudin-8 between bacterial treatments (Figure 3).

### 3.3. Confocal Laser Scanning Microscopy

Our previous study had demonstrated the presence of claudin-1, claudin-3, claudin-4, claudin-5, claudin-7, and claudin-8 in IPEC-J2 cells [24]. However, in IPEC-J2 cell layers cultured under the present conditions, only claudin-3 and claudin-4 showed continuous staining in TJs of all cells. In addition, occludin appeared to be a reliable TJ marker in these cell layers. In the present study, we therefore concentrated on the localization of claudin-3, claudin-4, and occludin.

**Figure 4:**
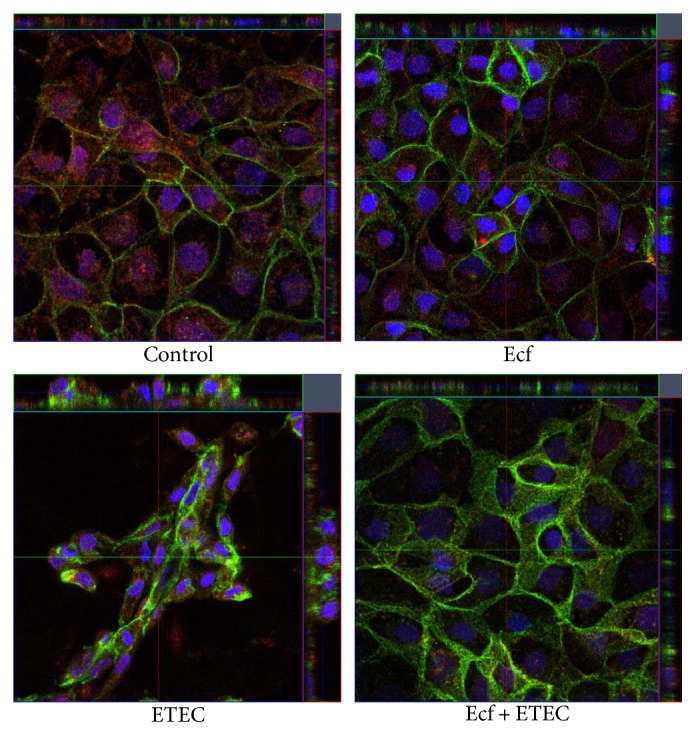
Confocal images of postconfluent IPEC-J2 cells at 6 h after treatment with bacterial strains (Ecf, ETEC, and Ecf + ETEC) and controls (red: claudin-3, green: claudin-4). Under all conditions, claudin-4 was localized in the tight junction and in the lateral membrane. *N* = 5 independent experiments.

**Figure 5:**
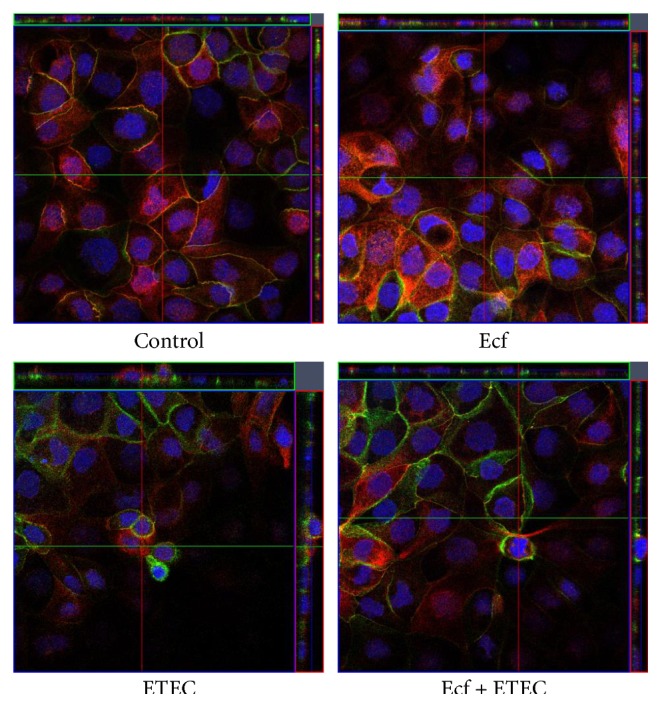
Confocal images of postconfluent IPEC-J2 cells 6 h after treatment with bacterial strains (Ecf, ETEC, and Ecf + ETEC) and controls (red: occludin, green: claudin-4). *N* = 5 independent experiments.

By CLSM, no redistribution of claudin-3, claudin-4, and occludin localization was observed under any experimental condition. However, for the first time, a reorganization of the epithelial layer was detected in the ETEC-incubated cells that was largely inhibited by coincubation with *E. faecium* (Figures 4 and 5).

### 3.4. Apoptosis and Cytotoxicity

**Figure 6:**
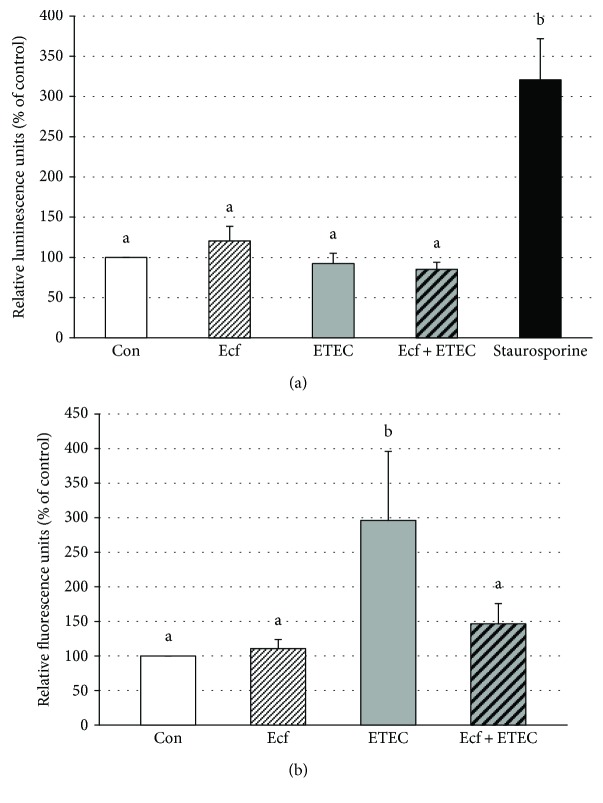
Apoptosis (a) and cytotoxicity (b) of IPEC-J2 cells after treatment with bacterial strains (Ecf, ETEC, and Ecf + ETEC) or without bacteria (Con = control) as assessed by caspase-3/7 activity in luminescence assay for apoptosis and by a dead cell protease fluorescence assay for cytotoxicity [means ± SEM]. Measurements were taken after 6 h. Groups differ significantly (*P* ≤ 0.05) when marked with different letters, (e.g. a, b) in the figure. *N* = 4 (apoptosis assay) and *N* = 5 (cytotoxicity assay) independent experiments.

To investigate the changes in the epithelial architecture detected by CLSM, the rate of apoptosis and cytotoxicity was examined. No significant differences were observed with regard to apoptotic events between the bacterial treatments of the cells (Figure 6(a)). However, cytotoxicity was significantly increased in the ETEC-incubated cells compared with either coincubated cells, *E. faecium-*incubated cells, or control cells (*p* ≤ 0.05) (Figure 6(b)). This represents a novel protective mechanism of *E. faecium* during ETEC infection.

### 3.5. Cytokine Expression

**Figure 7:**
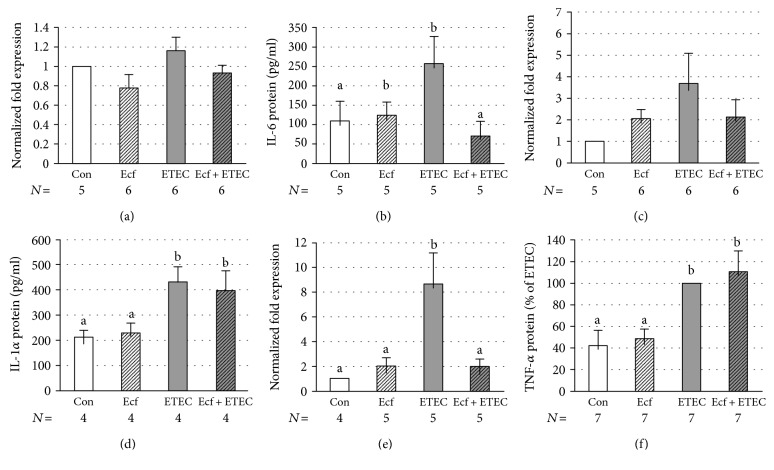
mRNA expression of IL-6 (a), IL-1*α* (c), and TNF-*α* (e) and cytokine release of IL-6 (b), IL-1*α* (d), and TNF-*α* (f) from IPEC-J2 cells after treatment with bacterial strains (Ecf, ETEC, and Ecf + ETEC) or without bacteria (Con = control) [means ± SEM]. Samples were taken after 4 h (mRNA) or 8 h (protein release). Groups differ significantly (*P* ≤ 0.05) when marked with different letters, (e.g. a, b) in the figure. *N* = number of independent experiments as indicated in the figure caption.

Since the epithelial barrier function can be influenced by inflammatory cytokines, such as TNF-*α* [25], the mRNA expression and release of several cytokines was assessed. IL-6 mRNA expression did not differ between bacterial treatments (Figure 7(a)). However, IL-6 protein release into the incubation medium was elevated in ETEC-incubated IPEC-J2 cells (*p* ≤ 0.05) (Figure 7(b)). This effect could be prevented when cells were coincubated with *E. faecium* (*p* ≤ 0.05) (Figure 7(b)).

IL-1*α* showed only a numerical increase in ETEC-incubated cells at the mRNA level, but a clear effect was detected at the protein level (*p* ≤ 0.05) (Figures 7(c) and 7(d)). The ETEC-induced increase of IL-1*α* protein was not reduced by coincubation with *E. faecium* (Figure 7(d)).

TNF-*α* was significantly upregulated at the mRNA level in cell lysates and at the protein level in the incubation medium when cells were incubated with ETEC (*p* ≤ 0.05) (Figures 7(e) and 7(f)). Increased release of TNF-*α* protein was not inhibited by coincubation with the probiotic strain; however, upregulation of TNF-*α* at the mRNA level was prevented when cells were coincubated with *E. faecium* (*p* ≤ 0.05) (Figures 7(e) and 7(f)).

### 3.6. cAMP ELISA

**Figure 8:**
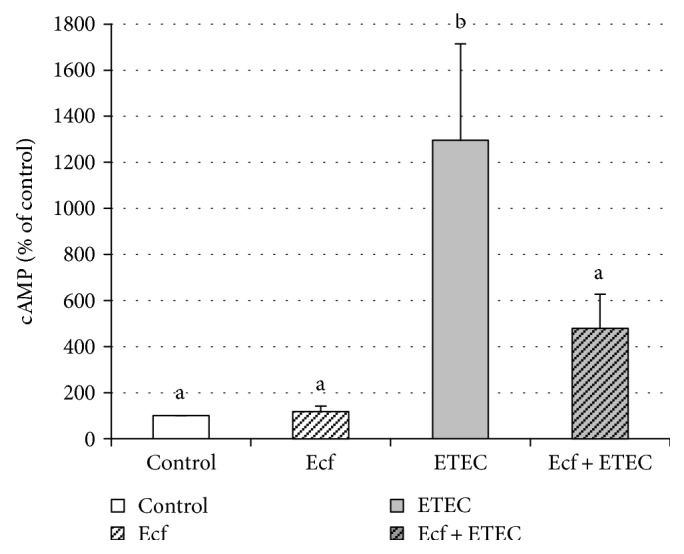
Intracellular cAMP concentrations in lysates of IPEC-J2 cells measured by ELISA at 4 h after commencement of incubation with bacterial strains (Ecf, ETEC, and Ecf + ETEC) compared with control [means ± SEM]. Groups differ significantly (*P* ≤ 0.05) when marked with different letters, (e.g. a, b) in the figure. *N* = 5 independent experiments.

Intracellular concentrations of cAMP were examined, since the heat-labile toxin of ETEC stimulates intracellular cAMP production, which can regulate the expression of proinflammatory cytokines [26]. Our data showed that the addition of ETEC increased the cAMP concentration in IPEC-J2 cells at 4 h after commencement of incubation with ETEC (*p* ≤ 0.05) (Figure 8). This increase was attenuated in cells coincubated with *E. faecium* (*p* ≤ 0.05) (Figure 8).

## 4. Discussion

In the present study, the effects of *E. faecium* during a challenge with ETEC were examined in a porcine intestinal epithelial cell culture model. The effects on epithelial cell function were analyzed by examining epithelial resistance, the expression of various TJ proteins, the epithelial cell structure, cytotoxic cell damage, and apoptosis. Furthermore, the release of inflammatory cytokines and intracellular cAMP concentrations was assessed.

One important step in revealing the underlying mechanisms of beneficial probiotics on epithelial barrier function is the investigation of changes in TJ protein expression and localization. We have shown recently that coincubation with *E. faecium* can reduce the effects of ETEC on *R*_t_ [15]. Since TJ proteins are crucial components of the intestinal barrier, changes at the TJ protein level appeared to be an attractive explanation for the probiotic effects detected. The intestinal epithelium represents a single epithelial cell layer which is interconnected by desmosomes, adherens junctions, and TJ proteins [27, 28]. TJs are the most apically located and determine the paracellular permeability. They can either seal the intercellular space or form selective paracellular pores [16]. The pathogenic ETEC strain has previously been shown to impair intestinal barrier integrity in IPEC-J2 cells through the loss of cell-cell contact, decreased cell viability, and a broken lining of the TJ adaptor protein zonula occludens-1 (ZO-1) [29] as well as changes in the expression and localization of claudin-1 [30, 31]. The present study has focused on claudin-1, claudin-3, claudin-4, and claudin-5, which are barrier-forming proteins, claudin-8 conveying a barrier for cations, and claudin-7, which is involved in changes in Cl^−^ and Na^+^ conductance [16, 32–34]. Intriguingly, in Western blots, no consistent differences in the expression levels of the barrier-sealing proteins claudin-1, claudin-3, claudin-4, claudin-5, and claudin-8 or of claudin-7 were found after bacterial treatment (Figure 3). This was unexpected, because ETEC produced profound effects on electrical tissue resistance (*R*_t_), and coincubation with *E. faecium* ameliorated and reversed this effect (Figure 2). A protective effect on *R*_t_ had also been observed in vitro for other probiotic strains such as *Lactobacillus plantarum*, which protected against the barrier disruption of intestinal epithelial cells challenged with ETEC [31]. Furthermore, treatment with *Lactobacillus casei* and *Butyricicoccus pullicaecorum* prevented a TNF-*α*-induced decrease in *R*_t_ in Caco-2 cells [35, 36]. As a novel finding, however, our data indicate that the protective effects of *E. faecium* on *R*_t_ are not based on changes in the expression of several TJ proteins but rather depend on changes in epithelial architecture.

Interestingly, in monolayers incubated with ETEC, a reorganization of the epithelial layer could be observed. Alterations in ETEC-infected cell layers ranged from single cells that protruded from the cell layer to dome formation or a stacked arrangement that seemed to compose a second epithelial cell layer in some areas. These cells were still attached to the main cell layer, as judged by their inclusion into an intact TJ pattern. The observed alterations are suggestive of increased cell shedding in the presence of ETEC. Epithelial rearrangement was greatly reduced or completely prevented in cells that had been preincubated with the probiotic strain before ETEC application revealing a novel protective probiotic mechanism during ETEC infection (Figures 4 and 5). Intestinal epithelial cell shedding and apoptosis can be induced, for example, by LPS or TNF-*α* [37–39]. To elucidate the underlying cellular mechanism, we examined the rate of apoptosis and cytotoxicity in the cells and the release of TNF-*α* and other cytokines. Our results revealed that differences in the epithelial structure are not attributable to apoptotic events but rather seem to depend on the cytotoxic effects of ETEC (Figure 6). In agreement with our findings, Lai et al. [40] have described increased cytotoxicity without apoptosis in ETEC-infected J774 macrophages. This commonly indicates that incubation with ETEC results in the necrosis of individual epithelial cells, an event that leads to localized barrier failure. Presumably, such focal necrosis and barrier failure is ameliorated and/or rapidly repaired in *E. faecium-*treated cells.

Since epithelial barrier function can be influenced by proinflammatory cytokines [25], changes in the cytokine response could further explain the finding that *E. faecium* prevents the ETEC-induced decrease in *R*_t_.

Cytokines are able to mediate communication between cells of the immune system, hematopoietic cells, and other cell types [41]. They are expressed not only by immune cells, but also by intestinal epithelial cells [42, 43]. In the present study, we have focused on a well-established set of inflammatory cytokines including IL-6, IL-1*α*, and TNF-*α* [44]. Assuming the probiotic *E. faecium* to have protective effects, we hypothesized that the probiotic strain would decrease the inflammatory cytokine expression caused by an ETEC infection. IL-6 is one of the most crucial inflammatory cytokines. However, IL-6 also has a protective function within the intestine. It prohibits epithelial apoptosis during ongoing inflammation and is necessary for epithelial proliferation [45]. In intestinal epithelial cells, IL-6 is required for the regeneration and for the maintenance of the barrier [46, 47]. At the TJ level, the release of IL-6 can lead to increased epithelial TJ permeability in Caco-2 cells [48–50]. On the other hand, the in vitro study of Suzuki et al. has revealed that claudin-1, claudin-3, and claudin-4 are not affected by IL-6 in Caco-2 cells. Our results indicate that the release of IL-6 is significantly reduced in coincubated cells compared with cells infected with ETEC alone (Figure 7(b)). Since IL-6 mostly functions as a proinflammatory cytokine, *E. faecium* might reduce inflammation during an ETEC infection. However, IL-6 might also be released in ETEC-incubated cells as a self-protective mechanism of the cells directed, for example, at the inhibition of apoptosis [45]. In either case, *E. faecium* seems to have a protective effect on the cells, as the stimulus for the release of IL-6 is inhibited by coincubation with this probiotic strain.

IL-1*α* exerts proinflammatory signals [51, 52] and is often released upon cell death [26]. It is physiologically attached to the cell nuclei, but translocates to the cytosol in the case of cell necrosis [53]. It provokes potent proinflammatory effects and is involved in neutrophil recruitment [52]. Moreover, IL-1 augments IL-8 expression and production in various cells [54]. In the present study, we could not detect any significant effects of the various bacterial treatments on the IL-1*α* mRNA level (Figure 7(c)). However, the release of IL-1*α* is significantly increased in ETEC-incubated and coincubated cells (Figure 7(d)). The latter indicates that the protective effects of *E. faecium* most likely do not depend on the modulation of IL-1*α* release.

TNF-*α* is an inflammatory cytokine that can promote apoptosis or the proliferation of cells [55]. Two forms of biologically active TNF-*α* are known: the membrane-bound and the soluble TNF-*α* [56]. In inflammatory bowel disease (IBD) patients, TNF-*α* is a crucial player that causes the loss of intestinal epithelial barrier integrity and stimulates the release of other proinflammatory cytokines [57]. TNF-*α* increases epithelial cell shedding, which can reduce intestinal barrier function [38, 58]. As such, TNF-*α* decreased *R*_t_ in T84 cells and Caco-2 cells [59, 60]. At the TJ level, TNF-*α* redistributes various TJ proteins [39, 61, 62]. In the present study, the mRNA expression of TNF-*α* is significantly increased 4 h after ETEC infection, whereas coincubation with *E. faecium* abolished this effect (Figure 7(e)). Wu et al. [31] obtained similar findings for TNF-*α* expression in IPEC-J2 cells incubated with the probiotic strain *Lactobacillus plantarum* and challenged with ETEC. Intriguingly, in our study, we could not detect a similar effect at the protein level (Figure 7(f)). The latter observation suggests that the release of TNF-*α* was rather small and could only be detected in the ELISA by increasing the sample volume to improve sensitivity. At such a low release level, the applied test may easily miss a possible decrease of TNF-*α* production when cells are coincubated with the probiotic strain (Figure 7(f)).

Cyclic AMP is a second messenger whose intracellular concentrations are adjusted by adenylate cyclase (AC) and cyclic nucleotide phosphodiesterase (PDE). AC heightens the level of cAMP, whereas PDE reduces its concentration in the cell. Cyclic AMP mainly activates protein kinase A (PKA), the GTP exchange protein directly activated by cAMP (EPAC) and cyclic nucleotide-gated ion channels [63]. Transcription factors modulating cytokine expression can be also regulated by cAMP [63]. Many triggers influence cAMP levels via AC and PDE, such as nutrients, hormones, neurotransmitters, pheromones, calcium, bicarbonate, CO_2_, and cAMP itself [64]. The heat-labile toxin (LT) released by ETEC stimulates AC, and the resulting increases in cAMP concentrations and PKA activity lead to opening of chloride channels such as cystic fibrosis transmembrane conductance regulator (CFTR) to induce intestinal fluid secretion and diarrhea [65, 66]. In the present study, the intracellular levels of cAMP are significantly increased in cells incubated with ETEC, thus indicating the release of LT by ETEC (Figure 8). When the cells are coincubated with *E. faecium*, however, the increase in cAMP concentrations is alleviated (Figure 8). The latter firstly indicates that *E. faecium* protects the cells at an early state of ETEC infection, since the release of LT, which upregulates the cAMP response, is one of the early events of ETEC pathogenesis upon its adherence to the intestinal mucosa.

## 5. Conclusions

The present study reveals that ETEC infection of IPEC-J2 cells leads to barrier failure via cytotoxic insults that trigger the necrosis of individual cells and subsequently alter epithelial architecture. Preexposure to the probiotic *E. faecium* has beneficial effects on subsequent ETEC infection via the downregulation of certain inflammatory cytokines, reduced cytotoxicity, and decreased concentrations of the second messenger cAMP. Our study has further confirmed results of a former investigation in which protective effects of *E. faecium* were observed on the *R*_t_ decrease induced by ETEC. We now demonstrate for the first time, however, that these effects cannot be attributed to changes in the expression levels of several tested TJ proteins but might be based on the ability of *E. faecium* to prevent cell death and to preserve the physiological epithelial cell structure in coincubated cells.

## Figures and Tables

**Table 1:** Oligonucleotide primers and amplicon length of PCR products.

Gene information	Primer sequence	Amplicon length	Accession number	Reference
*TNF*-*α* (tumor necrosis factor *α*, *Sus scrofa*)	(S) 5′-GCT GTA CCT CAT CTA CTC CC-3′	291 bp	NM_214022.1	[22]
	(AS) 5′-TAG ACC TGC CCA GAT TCA GC-3′			

*IL*-6 (interleukin 6, *Sus scrofa*)	(S) 5′-ATG CTT CCA ATC TGG GTT CA-3′	198 bp	M80258.1	
	(AS) 5′-GTG GTG GCT TTG TCT GGA TT-3′			

*IL*-1*α* (interleukin 1*α*, *Sus scrofa*)	(S) 5′-CAA GGA CAG TGT GGT GAT GG -3′	167 bp	NM_214029.1	
	(AS) 5′-TCA TGT TGC TCT GGA AGC TG-3′			

**Table 2:** Primary and secondary antibodies (WB = Western blot, IF = immunofluorescence).

*Primary antibodies*					
Antigen	Species	Company (catalog #)	Dilution WB	Dilution IF	
Claudin-1	Rabbit	Invitrogen/Thermo Fisher Scientific (51-9000)	1 : 1000		
Claudin-3	Rabbit	Invitrogen/Thermo Fisher Scientific (34-1700)	1 : 1000	1 : 200	
Claudin-4	Mouse	Invitrogen/Thermo Fisher Scientific (32-9400)	1 : 5000	1 : 200	
Claudin-5	Mouse	Invitrogen/Thermo Fisher Scientific (35-2500)	1 : 1000		
Claudin-7	Rabbit	Invitrogen/Thermo Fisher Scientific (34-9100)	1 : 2000		
Claudin-8	Rabbit	Invitrogen/Thermo Fisher Scientific (40-2600)	1 : 1000		
Occludin	Rabbit	Invitrogen/Thermo Fisher Scientific (71-1500)		1 : 200	
*β*-actin	Mouse	Sigma (A5441)	1 : 10,000		
*Secondary antibodies*					
Antigen	Species	Company (catalog #)	Conjugate	Dilution WB	Dilution IF
Mouse IgG	Goat	Jackson Immuno Research (115-225-146)	Cy2		1 : 600
Rabbit IgG	Goat	Jackson Immuno Research (111-175-144)	Cy5		1 : 600
Mouse IgG	Goat	Jackson Immuno Research (115-035-003)	HRP	1 : 10,000	
Rabbit IgG	Goat	Jackson Immuno Research (111-036-003)	HRP	1 : 10,000	

## References

[1] Di Mauro A., Neu J., Riezzo G. (2013). Gastrointestinal function development and microbiota. *Italian Journal of Pediatrics*.

[2] Kamada N., Seo S. U., Chen G. Y., Nunez G. (2013). Role of the gut microbiota in immunity and inflammatory disease. *Nature Reviews Immunology*.

[3] Hemarajata P., Versalovic J. (2013). Effects of probiotics on gut microbiota: mechanisms of intestinal immunomodulation and neuromodulation. *Therapeutic Advances in Gastroenterology*.

[4] Hold G. L., Smith M., Grange C., Watt E. R., El-Omar E. M., Mukhopadhya I. (2014). Role of the gut microbiota in inflammatory bowel disease pathogenesis: what have we learnt in the past 10 years?. *World Journal of Gastroenterology*.

[5] Matijasic M., Mestrovic T., Peric M. (2016). Modulating composition and metabolic activity of the gut microbiota in IBD patients. *International Journal of Molecular Sciences*.

[6] Kyriakis S. C., Tsiloyiannis V. K., Vlemmas J. (1999). The effect of probiotic LSP 122 on the control of post-weaning diarrhoea syndrome of piglets. *Research in Veterinary Science*.

[7] Zeyner A., Boldt E. (2006). Effects of a probiotic *Enterococcus faecium* strain supplemented from birth to weaning on diarrhoea patterns and performance of piglets. *Journal of Animal Physiology and Animal Nutrition (Berlin)*.

[8] Zhang L., Xu Y. Q., Liu H. Y. (2010). Evaluation of *Lactobacillus rhamnosus* GG using an *Escherichia coli* K88 model of piglet diarrhoea: Effects on diarrhoea incidence, faecal microflora and immune responses. *Veterinary Microbiology*.

[9] Wieler L. H., Ilieff A., Herbst W. (2001). Prevalence of enteropathogens in suckling and weaned piglets with diarrhoea in southern Germany. *Journal of Veterinary Medicine. B, Infectious Diseases and Veterinary Public Health*.

[10] Goswami P. S., Friendship R. M., Gyles C. L., Poppe C., Boerlin P. (2011). Preliminary investigations of the distribution of *Escherichia coli* O149 in sows, piglets, and their environment. *Canadian Journal of Veterinary Research*.

[11] van Beers-Schreurs H. M., Vellenga L., Wensing T., Breukink H. J. (1992). The pathogenesis of the post-weaning syndrome in weaned piglets: a review. *Veterinary Quarterly*.

[12] Scharek L., Guth J., Reiter K. (2005). Influence of a probiotic *Enterococcus faecium* strain on development of the immune system of sows and piglets. *Veterinary Immunology and Immunopathology*.

[13] Taras D., Vahjen W., Macha M., Simon O. (2006). Performance, diarrhea incidence, and occurrence of *Escherichia coli* virulence genes during long-term administration of a probiotic *Enterococcus faecium* strain to sows and piglets. *Journal of Animal Science*.

[14] Scharek-Tedin L., Kreuzer-Redmer S., Twardziok S. O. (2015). Probiotic treatment decreases the number of CD14-expressing cells in porcine milk which correlates with several intestinal immune parameters in the piglets. *Frontiers in Immunology*.

[15] Klingspor S., Bondzio A., Martens H. (2015). *Enterococcus faecium* NCIMB 10415 modulates epithelial integrity, heat shock protein, and proinflammatory cytokine response in intestinal cells. *Mediators of Inflammation*.

[16] Gunzel D., Fromm M. (2012). Claudins and other tight junction proteins. *Comprehensive Physiology*.

[17] Nusrat A., Turner J. R., Madara J. L. (2000). Molecular physiology and pathophysiology of tight junctions. IV. Regulation of tight junctions by extracellular stimuli: nutrients, cytokines, and immune cells. *American Journal of Physiology Gastrointestinal and Liver Physiology*.

[18] Bruewer M., Luegering A., Kucharzik T. (2003). Proinflammatory cytokines disrupt epithelial barrier function by apoptosis-independent mechanisms. *The Journal of Immunology*.

[19] Mankertz J., Tavalali S., Schmitz H. (2000). Expression from the human occludin promoter is affected by tumor necrosis factor alpha and interferon gamma. *Journal of Cell Science*.

[20] Hershko D. D., Robb B. W., Luo G., Hasselgren P. O. (2002). Multiple transcription factors regulating the IL-6 gene are activated by cAMP in cultured Caco-2 cells. *American Journal of Physiology. Regulatory, Integrative, and Comparative Physiology*.

[21] Lodemann U., Einspanier R., Scharfen F., Martens H., Bondzio A. (2013). Effects of zinc on epithelial barrier properties and viability in a human and a porcine intestinal cell culture model. *Toxicology in Vitro*.

[22] Conour J. E., Ganessunker D., Tappenden K. A., Donovan S. M., Gaskins H. R. (2002). Acidomucin goblet cell expansion induced by parenteral nutrition in the small intestine of piglets. *American Journal of Physiology Gastrointestinal and Liver Physiology*.

[23] Amasheh S., Meiri N., Gitter A. H. (2002). Claudin-2 expression induces cation-selective channels in tight junctions of epithelial cells. *Journal of Cell Science*.

[24] Zakrzewski S. S., Richter J. F., Krug S. M. (2013). Improved cell line IPEC-J2, characterized as a model for porcine jejunal epithelium. *PloS One*.

[25] Walsh S. V., Hopkins A. M., Nusrat A. (2000). Modulation of tight junction structure and function by cytokines. *Advanced Drug Delivery Reviews*.

[26] Yazdi A. S., Drexler S. K. (2013). Regulation of interleukin 1alpha secretion by inflammasomes. *Annals of the Rheumatic Diseases*.

[27] Ohland C. L., Macnaughton W. K. (2010). Probiotic bacteria and intestinal epithelial barrier function. *American Journal of Physiology and Gastrointestinal Liver Physiology*.

[28] Groschwitz K. R., Hogan S. P. (2009). Intestinal barrier function: molecular regulation and disease pathogenesis. *Journal of Allergy and Clinical Immunology*.

[29] Liu H. Y., Roos S., Jonsson H. (2015). Effects of *Lactobacillus johnsonii* and *Lactobacillus reuteri* on gut barrier function and heat shock proteins in intestinal porcine epithelial cells. *Physiological Reports*.

[30] Nassour H., Dubreuil J. D. (2014). *Escherichia coli* STb enterotoxin dislodges claudin-1 from epithelial tight junctions. *PloS One*.

[31] Wu Y., Zhu C., Chen Z. (2016). Protective effects of *Lactobacillus plantarum* on epithelial barrier disruption caused by enterotoxigenic *Escherichia coli* in intestinal porcine epithelial cells. *Veterinary Immunology and Immunopathology*.

[32] Alexandre M. D., Lu Q., Chen Y. H. (2005). Overexpression of claudin-7 decreases the paracellular Cl - conductance and increases the paracellular Na+ conductance in LLC-PK1 cells. *Journal of Cell Science*.

[33] Amasheh S., Milatz S., Krug S. M. (2009). Na+ absorption defends from paracellular back-leakage by claudin-8 upregulation. *Biochemical and Biophysical Research Communications*.

[34] Markov A. G., Falchuk E. L., Kruglova N. M., Rybalchenko O. V., Fromm M., Amasheh S. (2014). Comparative analysis of theophylline and cholera toxin in rat colon reveals an induction of sealing tight junction proteins. *Pflugers Archiv. European Journal of Physiology*.

[35] Eun C. S., Kim Y. S., Han D. S., Choi J. H., Lee A. R., Park Y. K. (2011). Lactobacillus casei prevents impaired barrier function in intestinal epithelial cells. *Acta Pathologica, Microbiologica, et Immunologica Scandinavica*.

[36] Eeckhaut V., Machiels K., Perrier C. (2013). *Butyricicoccus pullicaecorum* in inflammatory bowel disease. *Gut*.

[37] Williams J. M., Duckworth C. A., Burkitt M. D., Watson A. J., Campbell B. J., Pritchard D. M. (2015). Epithelial cell shedding and barrier function: a matter of life and death at the small intestinal villus tip. *Veterinary Pathology*.

[38] Watson A. J., Hughes K. R. (2012). TNF-alpha-induced intestinal epithelial cell shedding: implications for intestinal barrier function. *Annals of the new York Academy of Sciences*.

[39] Marchiando A. M., Shen L., Graham W. V. (2011). The epithelial barrier is maintained by *In Vivo* tight junction expansion during pathologic intestinal epithelial shedding. *Gastroenterology*.

[40] Lai X. H., Xu J. G., Melgar S., Uhlin B. E. (1999). An apoptotic response by J774 macrophage cells is common upon infection with diarrheagenic *Escherichia coli*. *FEMS Microbiology Letters*.

[41] Schooltink H., Rose-John S. (2002). Cytokines as therapeutic drugs. *Journal of Interferon and Cytokine Research*.

[42] Jung H. C., Eckmann L., Yang S. K. (1995). A distinct array of proinflammatory cytokines is expressed in human colon epithelial cells in response to bacterial invasion. *Journal of Clinical Investigation*.

[43] Stadnyk A. W. (2002). Intestinal epithelial cells as a source of inflammatory cytokines and chemokines. *Canadian Journal of Gastroenterology*.

[44] Callard R., George A. J., Stark J. (1999). Cytokines, chaos, and complexity. *Immunity*.

[45] Kuhn K. A., Manieri N. A., Liu T. C., Stappenbeck T. S. (2014). IL-6 stimulates intestinal epithelial proliferation and repair after injury. *PloS One*.

[46] Grivennikov S., Karin E., Terzic J. (2009). IL-6 and Stat3 are required for survival of intestinal epithelial cells and development of colitis-associated cancer. *Cancer Cell*.

[47] Schaper F., Rose-John S. (2015). Interleukin-6: biology, signaling and strategies of blockade. *Cytokine and Growth Factor Reviews*.

[48] Suzuki T., Yoshinaga N., Tanabe S. (2011). Interleukin-6 (IL-6) regulates claudin-2 expression and tight junction permeability in intestinal epithelium. *Journal of Biological Chemistry*.

[49] Ye D. M., Lei W. L., Al-Sadi R., Boivin M. A., Ma T. Y. (2012). IL-6 induced increase in intestinal epithelial tight junction permeability is mediated by AP-1 induced up-regulation of claudin-2 gene activity. *Gastroenterology*.

[50] Al-Sadi R., Ye D., Boivin M. (2014). Interleukin-6 modulation of intestinal epithelial tight junction permeability is mediated by JNK pathway activation of claudin-2 gene. *PloS One*.

[51] Palomo J., Dietrich D., Martin P., Palmer G., Gabay C. (2015). The interleukin (IL)-1 cytokine family—balance between agonists and antagonists in inflammatory diseases. *Cytokine*.

[52] Gresnigt M. S., van de Veerdonk F. L. (2014). The role of interleukin-1 family members in the host defence against *Aspergillus fumigatus*. *Mycopathologia*.

[53] Cohen I., Rider P., Carmi Y. (2010). Differential release of chromatin-bound IL-1 alpha discriminates between necrotic and apoptotic cell death by the ability to induce sterile inflammation. *Proceedings of the National Academy of Sciences of the United States of America*.

[54] Chaly Y. V., Selvan R. S., Fegeding K. V., Kolesnikova T. S., Voitenok N. N. (2000). Expression of IL-8 gene in human monocytes and lymphocytes: differential regulation by TNF and IL-1. *Cytokine*.

[55] Gaur U., Aggarwal B. B. (2003). Regulation of proliferation, survival and apoptosis by members of the TNF superfamily. *Biochemical Pharmacology*.

[56] Xu H., Uysal K. T., Becherer J. D., Arner P., Hotamisligil G. S. (2002). Altered tumor necrosis factor-alpha (TNF-alpha) processing in adipocytes and increased expression of transmembrane TNF-alpha in obesity. *Diabetes*.

[57] Slebioda T. J., Kmiec Z. (2014). Tumour necrosis factor superfamily members in the pathogenesis of inflammatory bowel disease. *Mediators of Inflammation*.

[58] Watson A. J., Duckworth C. A., Guan Y., Montrose M. H. (2009). Mechanisms of epithelial cell shedding in the mammalian intestine and maintenance of barrier function. *Annals of the new York Academy of Sciences*.

[59] Li Q., Zhang Q., Wang M. (2008). Interferon-gamma and tumor necrosis factor-alpha disrupt epithelial barrier function by altering lipid composition in membrane microdomains of tight junction. *Clinical Immunology*.

[60] Al-Sadi R., Guo S., Ye D., Ma T. Y. (2013). TNF-alpha modulation of intestinal epithelial tight junction barrier is regulated by ERK1/2 activation of Elk-1. *American Journal of Pathology*.

[61] Ma T. Y., Iwamoto G. K., Hoa N. T. (2004). TNF-alpha-induced increase in intestinal epithelial tight junction permeability requires NF-kappa B activation. *American Journal of Physiology and Gastrointestinal Liver Physiology*.

[62] He F., Peng J., Deng X. L. (2012). Mechanisms of tumor necrosis factor-alpha-induced leaks in intestine epithelial barrier. *Cytokine*.

[63] Fimia G. M., Sassone-Corsi P. (2001). Cyclic AMP signalling. *Journal of Cell Science*.

[64] Gancedo J. M. (2013). Biological roles of cAMP: variations on a theme in the different kingdoms of life. *Biological Reviews of the Cambridge Philosophical Society*.

[65] Nataro J. P., Kaper J. B. (1998). Diarrheagenic *Escherichia coli*. *Clinical Microbiology Reviews*.

[66] Kopic S., Geibel J. P. (2010). Toxin mediated diarrhea in the 21 century: the pathophysiology of intestinal ion transport in the course of ETEC, *V. cholerae* and rotavirus infection. *Toxins (Basel)*.

